# Editorial: Flexible Surgical Robotics: Design, Modeling, Sensing and Control

**DOI:** 10.3389/frobt.2022.854024

**Published:** 2022-04-19

**Authors:** Zheng Li, Long Wang, Liao Wu, Farshid Alambeigi, Shing Shin Cheng

**Affiliations:** ^1^ Department of Surgery, Multi-scale Medical Robotics Centre, Li Ka Shing Institute of Health Sciences, The Chinese University of Hong Kong, Hong Kong SAR, China; ^2^ Department of Mechanical Engineering, Schaefer School of Engineering & Science, Stevens Institute of Technology, Hoboken, NJ, United States; ^3^ School of Mechanical and Manufacturing Engineering, University of New South Wales, Sydney, NSW, Australia; ^4^ The Walker Department of Mechanical Engineering, University of Texas at Austin, Austin, TX, United States; ^5^ Department of Mechanical and Automation Engineering, The Chinese University of Hong Kong, Hong Kong SAR, China

**Keywords:** flexible surgical robot, continuum robot, soft actuator, surgical robot, catheter, endoscope

With the wide acceptance of minimally invasive surgeries due to their multitude of benefits over open surgeries in terms of reduced trauma, blood loss, and hospital stay, the surgical paradigm has been shifting to less or non-invasive approaches. Accessing the surgical site through natural orifices or minimal external incisions demands more flexibility, compliance, and distal dexterity in the surgical instruments to enable navigation through the tortuous anatomy, maintain clear visualization of the operating field, and offer tissue manipulation capability during the minimally invasive intervention. Conventional surgical instruments and devices are often not optimal for these minimally invasive procedures due to their straight and rigid structures. This gives rise to the development of flexible surgical robots, including snake-like flexible manipulators, continuum robots and soft robots.

Design, modelling, sensing, and control are four cornerstones of flexible surgical robot development. Compliance and payload are two critical yet contradictory requirements in the design of flexible surgical robots. Several methods have been proposed to improve the payload of flexible manipulators while maintaining their inherent compliance, including stiffness variable mechanisms and reinforced sheaths. Actuation is another important aspect in robot design. Common actuation methods of flexible surgical robots include cable-driven mechanisms, push-pull rod actuation, concentric pre-curved tubes, pneumatic/hydraulic actuation, smart materials, and magnetic forces. Cable-driven mechanisms are the most commonly used in flexible endoscopes and surgical instruments due to their simplicity, robustness, and large force transmission capability. Compared with cable-driven mechanisms that can only exert pulling actuation, elastic rods can provide both pulling and pushing actuation, potentially improving the stretch resistance and force/torque transmission. Actuation by concentric pre-curved tubes relies on the interaction among the pre-curved superelastic tubes *via* rotation and insertion of individual tubes. It enables the development of highly dexterous needle-like instruments but is subject to issues such as structural instability, limited curvature, and small distal payload. Pneumatic/hydraulic actuation is adopted by developing soft structures with fluid chambers, and the robot motion is typically determined by the structure design and fluid pressure. It could be especially appropriate as the actuators for robots that need to satisfy certain imaging compatibility (e.g., magnetic resonance imaging) requirements, but the issues of fluid leakage, cavitation, and response bandwidth must be taken into account. Smart materials, such as dielectric elastomer and shape memory alloy, typically offer large power density and strain under certain voltage or temperature change, and can also act as self-sensing components of flexible robots. However, the need for high actuation voltage and insufficient actuation bandwidth are features that need to be compromised or further improved. Compared to the previously mentioned actuation methods, magnetic force offers an effective wireless option that could be optimal to enable untethered flexible robot navigation, and has already been widely adopted in micro/nano robots. However, it suffers from limited actuation workspace, magnetic interference, and bulky electromagnetic actuation systems. While innovative approaches continue to be introduced to mitigate the problems and improve the performance of existing actuation methods, new materials, hybrid actuation, and novel design concepts, such as origami and growing structures, are being explored to increase the compactness and improve the motion capability of flexible surgical robots.

Modelling of flexible surgical robots mainly covers kinematic and static analysis. In kinematic modelling, a piecewise constant curvature assumption is commonly adopted. The flexible bending section is usually considered as a circular arc, and recent works also explore modal-based approaches to represent variable-curvature general shapes. For flexible manipulators, the constant curvature assumption is acceptable for no/neglectable payload and no internal friction conditions, while for soft robots, their own weight would make this assumption invalid. When external forces are applied to the flexible surgical robots, backbone deformation needs to be estimated by static modelling, in which Euler-Bernoulli beam theory and Cosserat rod theory are commonly used. Dynamic modelling of flexible surgical robots is difficult and related work is handful. Fortunately, flexible/soft surgical robots work with low speed. Friction compensation in actuation lines is another difficult problem that is usually coupled with the static modelling problem, which needs addressing in order to achieve instrument articulation accuracy.

Sensing in flexible surgical robots contains shape and interactive force with the environment. Shape of flexible robots determines the end-effector position and orientation. Unlike conventional robots with rigid links, in flexible robots, there is no joint and thus conventional joint encoders cannot be applied. Common approaches to estimate the shape of flexible robots include fiber Bragg grating (FBG) sensors, optical tracking, and electromagnetic sensors. In FBG sensors, axial strain is captured by the FBG wavelength and then used to calculate the deformation of the flexible robot backbone. Multiple fibers are required for complex shape sensing, and they need to be carefully encased. To characterize the deformation of flexible robots, optical tracking cameras can be used together with optical markers. This method suffers from line-of-sight problems and is usually used as the ground truth in experimental validation only. Considering flexible robots deform following spline curves, one can also use electromagnetic sensors to measure the position and orientation of endpoints of flexible manipulators and estimate the shape with spline curves. The shape of flexible robots is determined by the applied forces, including controlling forces and external interactive forces. Once the shape is measured, interactive forces with the environment can be derived from a static model. Another option is to develop dedicated force/haptic sensors to measure the contact forces directly.

Flexible surgical robots and soft robots usually work at low speeds. Their control mainly refers to kinematic control. Hysteresis and nonlinear deformation are common problems in accurate motion control of flexible/soft robots. In existing systems, while closed-loop control is usually applied at the motor actuation level, open-loop control *via* teleoperation is dominant at the system level, which relies on the surgeons to correct the errors in the camera view. Recent works have started exploring more autonomy using flexible robots despite the modelling and control difficulties. This requires closed-loop control at the system level, usually with visual feedback. Deep learning neural networks can be well adopted in analysing the video information and help to close the loop by combining with state-of-art control methods.

In this issue, three original articles have been included. Llyod et al. investigated the actuation of a soft continuum robot with magnetic force and proposed a fibre reinforcement method to reduce the unwanted twisting deformation of soft continuum manipulators by 67%. Donat et al. proposed a real-time shape estimation method for concentric tube continuum robots that involves a single force/torque sensor. In this work, the wrench at the tube base is used and combining with the discrete Kirchhoff rod theory, they were able to estimate the shape of the robot with up to 97% accuracy. Decroly et al. presented a soft robot for endoscopy developed using a scalable fabrication method. A 6 mm soft catheter containing three actuation chambers and a working channel is demonstrated for intubation in a bronchial model.

Flexible surgical robotics is a highly promising research area that could lead to technologies that not only significantly improve the clinical outcomes of surgical patients, but also make conventionally inoperable cases surgically treatable. The deployment of flexible surgical robots in the operating room for single port surgery, endoscopic surgery, bronchoscopy intervention, and vascular catheterization in the recent years is a testament to the fruitful outcome of nearly two decades of fundamental research in this area. It is hopefully a catalyst to high quality research towards the development and clinical adoption of more dexterous, miniaturized, and intelligent flexible surgical robots, benefiting both clinicians and patients.

